# Dengue, Zika and Chikungunya: Emerging Arboviruses in the New World

**DOI:** 10.5811/westjem.2016.9.30904

**Published:** 2016-09-29

**Authors:** Jessica Patterson, Maura Sammon, Manish Garg

**Affiliations:** Temple University Hospital and School of Medicine, Department of Emergency Medicine, Philadelphia, Pennsylvania

## Abstract

The arboviruses that cause dengue, chikungunya, and Zika illnesses have rapidly expanded across the globe in recent years, with large-scale outbreaks occurring in Western Hemisphere territories in close proximity to the United States (U.S.). In March 2016, the Centers for Disease Control and Protection (CDC) expanded its vector surveillance maps for *A. aegypti* and *A. albopictus*, the mosquito vectors for these arboviruses. They have now been shown to inhabit a larger portion of the U.S., including the heavily populated northeast corridor. Emergency physicians need to further familiarize themselves with these diseases, which have classically been considered only in returning travelers but may soon be encountered in the U.S. even in the absence of travel. In this paper, we discuss the presentation and treatment of dengue, Zika, and chikungunya, as well as special challenges presented to the emergency physician in evaluating a patient with a suspected arbovirus infection.

## INTRODUCTION

**Figure 1:**
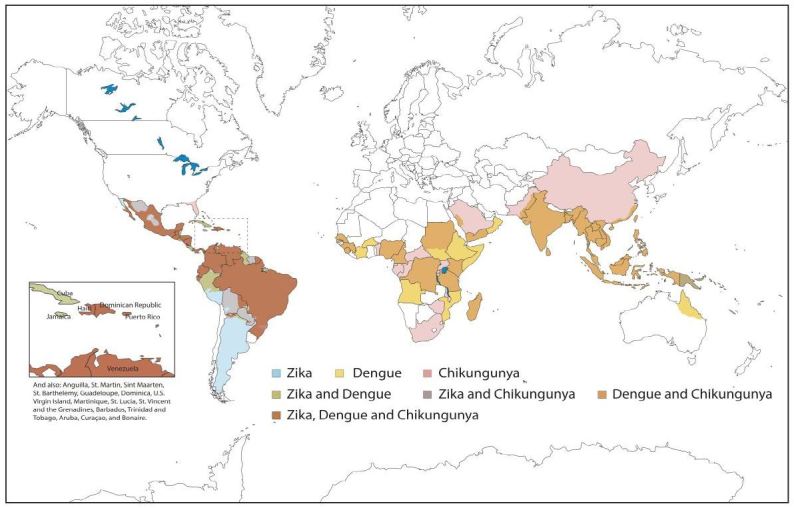
Map showing the estimated global distribution of dengue, Zika, and chikungunya.

With increases in globalization come increases in the spread of disease to populations lacking native immunity. One of the earliest known incidences of this phenomenon in the New World was the introduction of smallpox and syphilis to Native Americans during colonization. In recent years, emerging infectious diseases have been reported with greater frequency. In 1999, West Nile Virus was first reported in New York^1^ and quickly became endemic throughout the United States (U.S.). Local transmission of dengue occurred in Florida in 2009,^2^ and in 2013 and 2014 a chikungunya epidemic spread rapidly through South America and the Caribbean.^3^ We are now faced with a pandemic of Zika virus, which is quickly spreading through the tropical areas of the Western Hemisphere, with growing concerns that an outbreak could soon occur in the mainland U.S. Yellow fever is another important arbovirus transmitted by *Aedes* mosquitos, though an effective vaccine exists and massive vaccination campaigns in South America have prevented large-scale outbreaks in the Western Hemisphere during this century.^4^ Emergency physicians (EP) are on the front lines of detection and treatment of these illnesses, though due to their rarity, many clinicians are unfamiliar with these disease processes. EPs must be vigilant in eliciting a careful travel history in any febrile patient and should not rely on basic triage screening. Given that vector-borne illnesses are endemic in virtually every region of the world, a positive travel history should prompt consideration of diagnoses including malaria, arboviruses, and other tropical infections. In this article, we will review the vectors, the diagnoses, and treatments of three of the most rapidly spreading arboviruses in the Western Hemisphere: dengue, Zika, and chikungunya^5^–^8^ (Figure 1).

## THE VECTORS

Mosquitos from the genus *Aedes*, specifically *A. aegypti* and *A. albopictus*, are responsible for the transmission of many arboviruses worldwide. Despite their likely initial origins as zoonoses, humans have become the primary amplifying host of these viruses, particularly in urbanized settings.^9^ Transmission occurs when a mosquito bites an infected individual and then directly carries the virus to another person.

The *A. aegypti* mosquito has traditionally been considered to be a much more efficient vector for the spread of these diseases due to several factors. *A. aegypti* has evolved to live its entire life cycle from larvae to adult in close proximity with its human hosts, strongly preferring to feed on humans even in the presence of other mammals. *A. aegypti* will bite several humans in the course of a single blood meal. This behavior can rapidly transmit a virus to multiple hosts in a short time frame, efficiently propagating disease. *A. albopictus* lacks this highly preferential coexistence with humans, living in a more varied environment. Because they feed on other dead-end hosts (i.e. dogs, cats, squirrels), this provides a hindrance to rapid disease amplification. *A. albopictus* has a greater tolerance for cold environments, and thus while it is a less efficient vector of disease, it can pose a threat to a larger geographic area.^10^

**Figure 2a,b:**
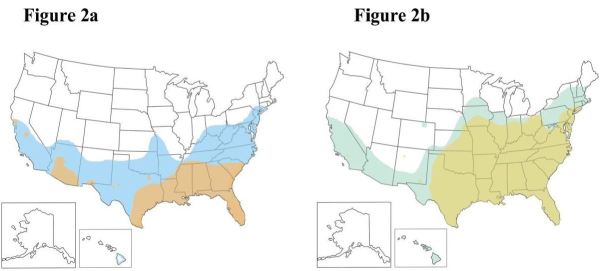
In April 2015, the CDC updated its vector surveillance maps to depict that both *A. aegypti* and *A. albopictus* are now believed to inhabit a wider range of distribution in the U.S. Figure 2a illustrates the expansion of territory covered by *A. aegypti*; Figure 2b illustrates the expansion of *A. albopictus*. The range of both mosquitos has spread significantly to the north and west. Although the significance of this expansion in epidemiologic terms is unclear, it may place a greater proportion of the population at risk for exposure to emerging arboviruses such as Zika, particularly during warmer months.

The transmission of these arboviruses in the Western Hemisphere was delayed by aggressive vector control campaigns in the 1960s and 1970s. However, these efforts have since lapsed, facilitating spread of the mosquitoes.^11^ These mosquitoes have subsequently grown in their distribution in the U.S., and in March 2016 the Centers for Disease Control and Prevention (CDC) updated its vector maps to reflect this spread. *A. aegypti*, the more concerning of these vectors, is now suspected to inhabit the heavily populated northeast corridor^12^ (Figure 2). This has increased concern among public health experts that these diseases may emerge in the continental U.S. in a more widespread fashion. Traditionally, EPs in the U.S. have considered these diseases only in the returning traveler; in the near future, however, dengue, Zika, and chikungunya may need to be considered in the absence of recent travel.

## THE ARBOVIRUSES

### Dengue

#### Background and Clinical Course

Dengue is the most prevalent and dangerous of the emerging arboviruses. According to the World Health Organization (WHO), it is the most rapidly spreading arbovirus worldwide and is endemic in every inhabited region of the world except for continental Europe.^13^ A 2013 study estimated that 96 million clinically significant cases occur annually, a dramatic increase from 50 million in 2009.^14^ Although most cases in the U.S. have been in returning travelers, sporadic outbreaks have occurred in Louisiana, Hawaii, Florida, and Texas.^15^

Dengue is a member of the flavivirus genus, which also includes yellow fever, West Nile, and Zika viruses. There are four distinct dengue virus serotypes, with type 2 considered to be the most virulent strain. Although the human-mosquito-human transmission cycle is the most prominent method of propagation, dengue can be transmitted vertically during pregnancy and via blood-borne transmission. Dengue is not transmitted via sexual contact or respiratory droplets.^5^,^16^

**Figure 3:**
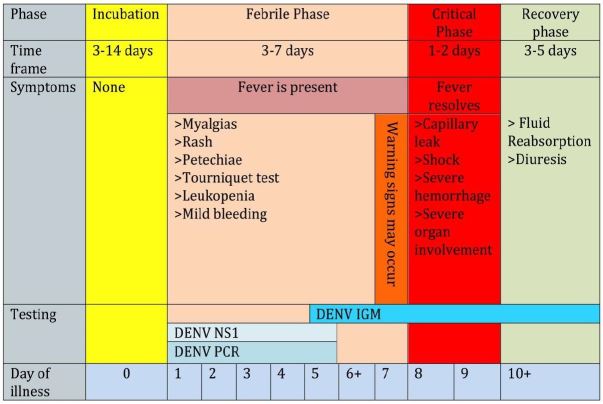
Three distinct phases of dengue infection have been described: incubation, febrile, and recovery. The critical phase, when patients may become unstable, typically occurs after defervescence of the fever. Although most patients will improve after the febrile stage, those who progress to the critical phase may display warning signs. By closely monitoring for these signs, clinicians can identify and appropriately disposition patients at higher risk for a more severe clinical course. The laboratory evaluation of dengue also varies based on the stage of infection and thus samples evaluating for both viral practices (PCR or NS1) and IgM levels should be ordered. *DENV,* dengue virus; *NS1,* nonstructional protein 1; *PCR,* polymerase chain reaction *PCR is expressed on the surface of infected cells.

About 50% of dengue infections are symptomatic. The clinical presentation of dengue illness is widely varied and its course unpredictable, making diagnosis and treatment challenging. There are three distinct phases of symptomatic dengue that have been well described: febrile, critical and recovery^13^,^16^ (Figure 3).

#### Febrile Phase

The febrile phase typically lasts 2–7 days, with nonspecific symptoms such as myalgias, arthralgias, headache, rash, nausea and vomiting.^17^ The rash can range from a mild erythema to a pruritic, macular rash with small circular islands of spared skin classically described as “isles of white on a sea of red.” Minor hemorrhagic manifestations such as petechiae and epistaxis may occur. Laboratory findings are nonspecific and can include hyponatremia, leukopenia, thrombocytopenia, and transaminitis.^13^,^16^,^18^

The majority of symptomatic patients will improve after the febrile stage. However, about 5% will progress to the critical phase, which occurs after the virus is cleared from the bloodstream and the fever resolves.^19^ The absence of fever should not be reassuring to clinicians, as patients can rapidly deteriorate after defervesence.^13^

#### Critical Phase

The critical phase, which lasts 1–2 days, is marked by an increase in capillary permeability, thrombocytopenia, and possible progression to hemorrhage. Leaky capillaries lead to a loss of plasma volume, and its presentation can range from mild edema to pleural effusions, ascites and shock with end-organ damage. A severe hemorrhagic diathesis requiring transfusion may occur. Significant hemorrhage and capillary leak syndrome can occur concurrently or be independent of each other.^20^

#### Recovery Phase

The recovery phase, which lasts 3–5 days, occurs when the patient stabilizes and reabsorbs extravasated fluid. New complications may develop, including acute pulmonary edema, which can occur in the setting of excessive intravenous fluid (IVF) resuscitation.^13^

#### Challenges for the Emergency Physician: Diagnosis and Disposition

**Figure 4:**
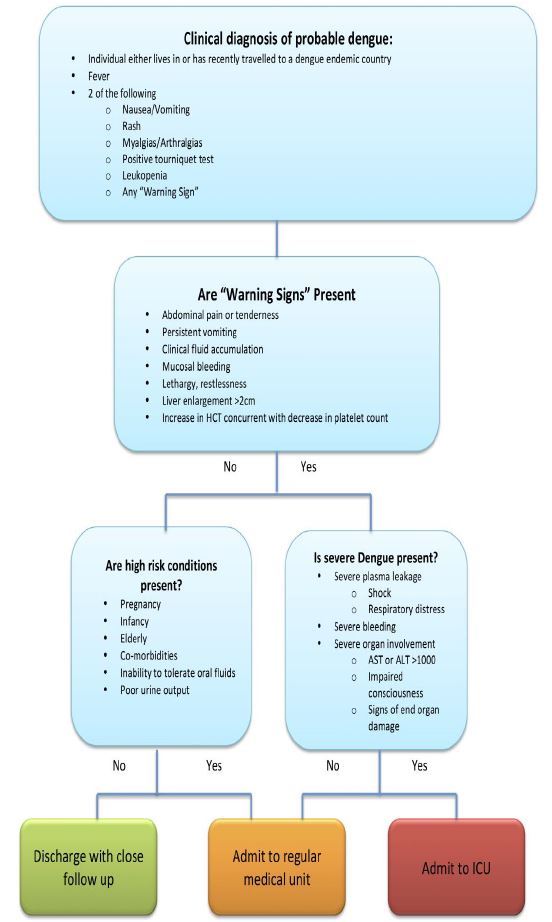
Management algorithm for dengue, adapted from Dengue Case Management, available at: http://www.cdc.gov/dengue/resources/DENGUE-clinician-guide_508.pdf. *HCT*, hematocrit; *AST*, aspartate amino transferase; *ALT*, amino alanine transferase; *ICU*, intensive care unit AST/ALT values are in units/liter.

While the EP may not ultimately make the definitive diagnosis of dengue, it is important that they both consider the diagnosis in the appropriate patient, and determine which patients are at risk for a poor outcome and thus warrant admission. Left untreated, severe dengue carries a mortality rate of 20% that if properly managed can be reduced to less than 1%.^21^ Thus, early recognition is crucial. Dengue should be considered in any symptomatic patient presenting within two weeks of returning from an endemic area. The most recent WHO guidelines published in 2009 are directed toward early recognition of susceptible patients and use a clear algorithm^13^ (Figure 4). In these new guidelines, the previously used classifications of dengue fever, dengue hemorrhagic fever, and dengue shock syndrome have been replaced by the terms dengue without warning signs, dengue with warning signs, and severe dengue. This updated classification was designed to help clinicians make disposition decisions, and is thus particularly useful in the emergency department (ED). This revised classification system has shown increased sensitivity for identification of severe cases.^22^

Once considering dengue, the emergency medicine clinician must decide whether the patient is at risk of progressing to severe dengue. The WHO has identified both “risk factors” by history or demographics that make a patient more susceptible to severe dengue as well as clinical “warning signs” that signify deterioration towards the more dangerous critical phase.^13^ When determining whether or not to admit a patient with suspected dengue, both must be considered.

**Figure 5:**
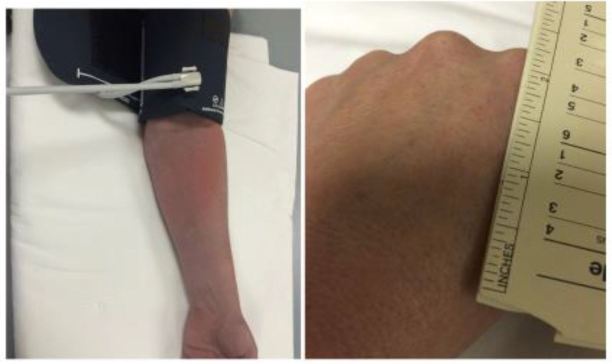
The tourniquet test, which is a marker of capillary fragility, is a quick and easy bedside study that can help physicians differentiate dengue from other illnesses, although it lacks both sensitivity and specificity. A blood pressure cuff is inflated to midway between the systolic and diastolic blood pressure and maintained for five minutes. A positive test is the presence of 10 or more petechiae per square inch. 16,19.

The WHO favors a clinical diagnosis of “probable dengue” for any patient who lives in or has traveled to a dengue endemic area and has a history of fever and any two of the following: nausea/vomiting, rash, aches and pains, positive tourniquet test, (Figure 5) leukopenia, or “any warning sign.”^13^,^23^ Further information on the laboratory evaluation of dengue can be found in the testing section of this article.

#### Risk Factors

Patients with risk factors such as pregnancy, chronic comorbidities (i.e. diabetes, organ failure, immunosuppression), and extremes of age are more likely to develop severe dengue and should be admitted, even if symptoms are mild.^13^ Despite evidence that infection with one dengue serotype confers lifelong immunity against that serotype, it does not confer long-lasting protection against the other serotypes. It is in fact critical that the EP recognize a unique phenomenon of dengue: previous infection with a different serotype can paradoxically increase the risk for development of severe dengue.^16^ The prevailing hypothesis for this phenomenon is dengue antibody-dependent enhancement (ADE). According to this hypothesis, circulating IgG antibodies form complexes with the virus during active infection, promoting uptake of the virus by macrophages where the virus replicates.^24^,^25^ Consequently due to ADE, as the incidence of dengue continues to increase, clinicians may see more patients who have been re-infected with dengue and thus have more severe presentations with increased fatalities.^25^

#### Warning Signs

Patients will often display warning signs of severe dengue prior to progression to the critical phase. The following warning signs identify patients who may be progressing towards severe dengue: abdominal pain or tenderness, persistent vomiting, clinical fluid accumulation, mucosal bleed, lethargy/restlessness, or liver enlargement > 2cm. An increasing hematocrit is also seen as a warning sign - as the plasma leaks into the extravascular spaces, hematocrit increases, signifying intravascular dehydration.^13^,^20^ Recognition of these signs can be life saving.

The treatment of dengue is supportive and based on clinical stage and presence of warning signs. Patients should not be given aspirin or other non-steroidal anti-inflammatory drugs (NSAIDs) as they may complicate hemorrhage.^13^ Neither are steroids recommended.^26^

A patient with no risk factors and no warning signs can be discharged if they are well appearing, tolerating oral intake, and are producing good urine output.^13^ In the febrile phase, only dehydrated patients or those not taking adequate oral intake should receive intravenous fluids (IVF). Reliable short-term follow up must be ensured before discharging a patient; the CDC and WHO recommend daily follow-up visits through the critical period.^27^

Patients with any warning sign present should be admitted for observation and supportive care as the clinical deterioration in the critical phase can often occur rapidly. In the critical phase, IVF should be administered to maintain a urine output of at least 0.5 milliliters per kilogram per hour; however, excessive fluids can worsen plasma leakage.

Patients with severe dengue require admission to an intensive care unit for supportive care and monitoring. Severe dengue is present if any of the following are met: severe plasma leakage resulting in shock and/or fluid accumulation with respiratory distress; severe bleeding as evaluated by the clinician; or signs of severe organ involvement (i.e. aspartate transaminase (AST) or alanine transaminase (ALT) >1000, impaired consciousness, etc.). Early signs of plasma leakage include tachycardia and a narrowed pulse pressure. Transfusion of blood products should be driven by clinical presentation if needed.^28^

### Zika

#### Background and Clinical Course

Zika virus, named after the Ugandan forest in which it was discovered, is a flavivirus closely related to dengue. It was first isolated in 1947 in a macaque monkey and shortly thereafter was recognized to cause an asymptomatic infection or mild febrile illness in humans. For decades, Zika was of little concern to clinicians. However, since a correlation between Zika virus infection and fetal microcephaly was discovered, Zika has received significant public health and media attention.^29^ In February 2016, the WHO officially declared it a “Public Health Emergency of International Concern”.^30^

Zika was relatively unknown outside small outbreaks in Africa and Southeast Asia until 2007, when a large outbreak occurred in Yap, a small island in Micronesia. According to serological data, 73% of the island’s population was infected during the outbreak.^31^ Outbreaks were subsequently noted to occur across the Pacific islands before eventually emerging in the Western Hemisphere in March 2015, when Brazil reported the first case of Zika virus in the Americas. By December 2015, Brazil suspected 1.3 million cases of Zika virus; by April 2016 Zika virus had spread to 33 countries and/or territories^32^ (Figure 1). Until the recent reports of local Zika transmission in southern Florida, all cases reported in the U.S. had been linked to returning travelers or their sexual partners.^33^ It is unknown at the time of this writing to what extent this disease will spread throughout the U.S.

Zika virus is spread by several mosquito species worldwide, but *Aedes* species are responsible for most outbreaks.^32^ Although the primary mechanism of transmission is via an infected mosquito, there have been cases of sexual transmission to partners of returning travelers.^34^,^35^ Blood-borne transmission is likely possible during the viremia stage, and transfusion-related infections have been reported in Brazil. It has also been isolated in urine, saliva, and breast milk of infected individuals, though no transmission from these sources have been identified to date.^36^,^37^

**Figure 6:**
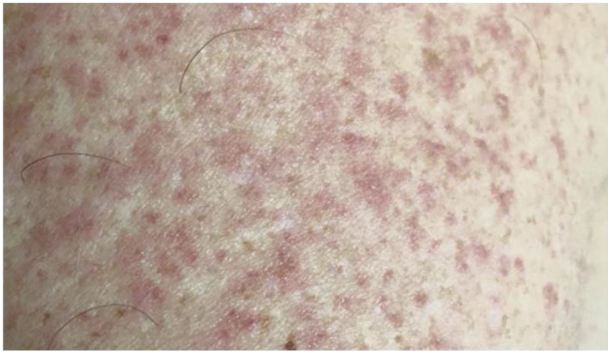
Rash on a patient with Zika infection.

Once infected, the incubation period of Zika virus is not yet clearly defined but currently presumed to be less than two weeks. During viremia a mild illness can develop with symptoms such as fever, nonpurulent conjunctivitis, a maculopauplar rash (Figure 6)*,* arthritis/arthralgias, headache, and vomiting.^32^ Severe disease and complications requiring hospitalization are uncommon, and it has not been shown to cause a severe capillary leak syndrome or hemorrhagic fever.^36^ It is estimated that up to 80% of infections are asymptomatic.^38^ Like dengue, treatment is supportive. The symptoms are clinically indistinguishable from the febrile stage of dengue, so aspirin, NSAIDs and steroids should be avoided.^39^

Although the mechanism is not yet understood, a strong link has been established between maternal Zika virus infection and serious birth defects including microcephaly and other serious brain malformations.^40^ Zika virus has been found in the amniotic fluid, brain tissue, and placenta of infants born with cerebral abnormalities during outbreaks,^37^ and a proposed mechanism has been suggested.^41^ Although there have been concerns that the correlation was inflated from over-reporting during outbreaks, in April 2016 the WHO declared a “scientific consensus that Zika virus is a cause of microcephaly.”^42^ The risk of fetal malformation is presumed to be highest when maternal infection occurs in the first trimester,^38^ though adverse pregnancy outcomes have been associated with infections in all trimesters.^43^

#### Challenges for the Emergency Physician: Counseling Patients

The majority of patients with suspected Zika virus would be safe for discharge home from the ED. Thus, the EP should be prepared to directly counsel patients with suspected Zika virus regarding the risks of direct transmission and potential complications from the infection.

#### Women of Childbearing Age

In particular, women of childbearing age and pregnant women with suspected Zika should be counseled regarding the potential for birth defects. The CDC currently recommends that pregnant women avoid travel to areas affected by Zika. If travel cannot be avoided, strict mosquito protection precautions should be taken. Current recommendations for pregnant patients with Zika infection during pregnancy include serial ultrasounds performed every 3–4 weeks.^38^ In light of this, pregnant patients with suspected Zika should have short-term obstetric follow up arranged prior to discharge.

Women with suspected Zika virus should wait at least eight weeks after the onset of their symptoms to have unprotected sex.^38^ Prior infection with Zika virus is not a risk factor for birth defects; the increased risk is associated with active viremia.^44^ Breast-feeding patients should be warned that Zika virus has been detected in breast milk, although no cases of transmissions have been reported to date.^37^

#### All Patients

The CDC currently recommends that all patients with suspected Zika virus should refrain from unprotected sex with women for six months. Individuals who have travelled to an endemic area, but did not develop symptoms should refrain for at least eight weeks after return.^45^ The time duration of potential risk from sexual transmission has not yet been confirmed, but viral particles have been detected in semen as long as 62 days after the onset of symptoms.^46^

Also of concern to the EP is the correlation between Zika virus infection and Guillain-Barre Syndrome (GBS). Several countries in the western Pacific and Americas have reported increases in GBS during outbreaks.^32^ One case control study in French Polynesia reported an odds ratio of greater than 34 between previous Zika virus infection and GBS.^32^ It is reasonable to counsel patients with suspected Zika of this potential risk so that they know to seek medical attention at the first symptoms of GBS.

Patients should also be counseled on responsible behavior to avoid spread of Zika virus via local mosquito vectors. Due to the high incidence of asymptomatic infection, the current CDC recommendations are that all patients wear mosquito repellant for three weeks after return from a Zika-infected area to prevent local transmission, particularly in areas with reported *Aedes* activity.^47^

### Chikungunya

#### Background and Clinical Course

Chikungunya, an alphavirus of the *Togaviridae* family, is a mosquito-spread virus that causes a febrile illness characterized by severe arthralgias.^48^,^49^

Chikungunya was first isolated in Tanzania in 1953 in a febrile patient. It is named after a word in the local *Makonde* dialect that roughly translates to “that which bends up,” referring to the stooped position patients with severe joint pain often develop.^50^ For 20 years, it was a rarely reported disease; however, beginning in 2004 large-scale outbreaks were noted to occur throughout Africa and Asia.^51^ The first case of chikungunya in the Western Hemisphere was reported in 2013; by December 2015 it had rapidly spread to 44 countries and territories.^51^,^52^ Studies of these epidemics found a notably high infectivity rate, ranging from 34–45%.^53^ In 2014 chikungunya was locally transmitted in the U.S., with 11 cases reported in Florida.^54^ (Figure 1)

The clinical presentation of chikungunya is similar to dengue and Zika; however, in contrast to these other diseases, the majority of people infected with chikungunya are symptomatic.^49^ After an incubation period ranging from 1–12 days (typically 3–7 days), viremia occurs, and symptoms develop. The fever is typically high grade with a sudden onset. Arthralgia is present in nearly all cases and can be disabling. The pain is typically symmetric, worse in the morning, relieved by mild exercise but worsened by strenuous activity. The most common joints involved are ankles, wrists, and fingers. Migratory polyarthritis with effusions can also occur.^49^,^55^ In about half of patients a maculopapular rash will develop, occasionally with vesiculobullous eruptions and ulcers.

As with the other illnesses discussed here, treatment for chikungunya is supportive. Acetaminophen is preferred for pain and fever control over NSAIDs due to its similarity in presentation with, and possible misdiagnosis of dengue.^49^ Admission will rarely be required. During past epidemics, patients with symptoms severe enough to warrant admission typically have comorbid conditions. Neurologic complications including meningo-encephalitis, seizures, and acute encephalopathy have been reported; such manifestations occur more often in children than adults.^48^,^56^ Vertical transmission of chikungunya has been reported and is associated with a more severe presentation in the neonate.^49^

#### Challenges for the Emergency Physician: Long-term Complications

Most patients will have resolution of their symptoms in 1–3 weeks. However, chikungunya is unique in that severe arthralgias may persist for months to years. The WHO estimates this cohort to be about 10%,^55^ but a study of the Reunion Island epidemic found that more than half of patients reported either recurrent or persistent rheumatic symptoms 15 months after infection. Risk factors for persistent symptoms include age over 45, comorbid conditions (i.e. diabetes, organ failure, immunosuppression), and severity of pain at the onset of symptoms.^57^ The mechanism is not well understood, but may be linked to persistent circulating IgM antibodies.^58^ Based on radiographic findings of erosive arthritis in joints of patients one year after infection, there is evidence that chikungunya infection may be a precursor to development of rheumatoid arthritis.^59^,^60^ Persistent joint pain from chikungunya infection may respond to NSAIDs and should be the first-line therapy in the absence of contraindications.^49^,^55^ Patients discharged from the ED with suspected chikungunya should be counseled regarding these known complications.

## IDENTIFYING AND DIAGNOSING THE UNDIFFERENTIATED PATIENT

A thorough travel history should be obtained in all patients, particularly those presenting with febrile illness. In the ED the diagnosis of dengue, Zika, and chikungunya should all be made on clinical grounds. Given the long turnaround time, serum testing has no role in emergent management. However, given the significant overlap in clinical presentation and vector locations, particularly in the early stages of presentation, consideration of one of these entities should lead to further evaluation of the other two. The CDC, on both clinical and epidemiologic grounds, recommends this approach.^61^

Once malaria has been excluded, it is prudent to assume dengue as the leading differential diagnosis when determining a disposition in a patient who has traveled to an endemic area. Because dengue can progress quickly from a simple viral illness to a life-threatening condition, the search for dengue warning signs should always be considered even when Zika or chikungunya is strongly suspected. In all such patients, acetaminophen is preferred for pain and fever control over NSAIDs given the risk for hemorrhagic complications in dengue.

All patients who are discharged from the ED should be counseled on the potential complications associated with Zika and chikungunya until a definitive diagnosis has been determined.

**Figure 7:**
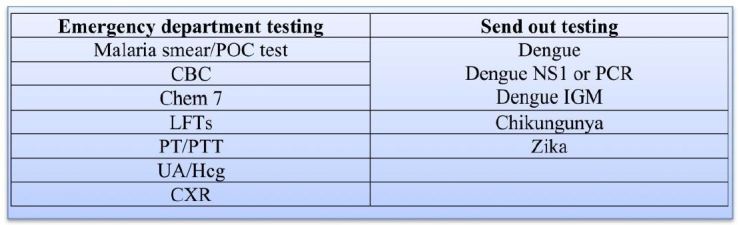
Proposed testing algorithm for the initial evaluation of a patient with suspected arbovirus infection. Depending on the region of travel, malaria and other native pathogens such as typhoid and leptospirosis also should be considered. *POC*, point of care; *CBC*, complete blood count; *LFT*s, liver function tests, *PT/PTT*, prothrombin time/partial thromboplastin time; *UA/Hcg*, urinalysis/human chorionic gonadotropin, *CXR*, chest x-ray; *PCR*, polymerase chain reaction

All three of these entities are considered reportable conditions. Although commercial laboratory testing is available for dengue, Zika and chikungunya, most testing for Zika is currently done only through the CDC or state health departments. We recommend the following testing algorithm (Figure 7)*.* If a patient has travelled to an area endemic for malaria, we strongly recommend malaria testing as well. Other illnesses such as yellow fever, typhoid, leptospirosis, and helminth infections should be considered on an individual basis if indicated by the travel history. Please refer to the CDC website for up-to-date testing recommendations, as guidelines may change due to the shifting nature of this pandemic.

## PERSONAL PROTECTION IN ENDEMIC AREAS

The importance of personal protection in endemic areas cannot be emphasized enough, both as an issue to personal safety as well as a public health measure. The CDC currently recommends the use of an Environmental Protection Agency-registered insect repellant such as DEET or picaridin. These agents have been proven to be safe and effective for use in infants over the age of two months, and in pregnant or breastfeeding women. A list of approved agents can be found at https://www.epa.gov/insect-repellents. The treatment of clothing with permetherin is also recommended, and has been shown to be safe and effective.^62^ Additional behavioral methods, such as the use of screens and mosquito netting, are also important.

The extent of the progression of the Zika epidemic into the continental U.S. remains uncertain at this time, but increasing globalization has weakened the traditional barriers that once contained diseases within regions. Patients with these novel diseases are likely to present first to the ED. Prompt recognition and treatment of these diseases will lead to both better provisions of care to individual patients, as well as assistance to public health officials with containing these outbreaks.
